# How Implementation of Systems Biology into Clinical Trials Accelerates Understanding of Diseases

**DOI:** 10.3389/fneur.2014.00102

**Published:** 2014-06-27

**Authors:** Bibiana Bielekova, Yoram Vodovotz, Gary An, John Hallenbeck

**Affiliations:** ^1^Neuroimmunological Diseases Unit, National Institute of Neurological Disorder and Stroke, National Institutes of Health, Bethesda, MD, USA; ^2^Center for Human Immunology of the National Institutes of Health, Bethesda, MD, USA; ^3^Department of Surgery, University of Pittsburgh, Pittsburgh, PA, USA; ^4^Center for Inflammation and Regenerative Modeling, McGowan Institute of Regenerative Medicine, Pittsburgh, PA, USA; ^5^Department of Surgery, Northwestern University, Chicago, IL, USA; ^6^Stroke Branch, National Institute of Neurological Disorder and Stroke, National Institutes of Health, Bethesda, MD, USA

**Keywords:** systems biology, clinical trials, clinical trials methodology, multiple sclerosis, polygenic diseases

## Abstract

Systems biology comprises a series of concepts and approaches that have been used successfully both to delineate novel biological mechanisms and to drive translational advances. The goal of systems biology is to re-integrate putatively critical elements extracted from multi-modality datasets in order to understand how interactions among multiple components form functional networks at the organism/patient-level, and how dysfunction of these networks underlies a particular disease. Due to the genetic and environmental diversity of human subjects, identification of critical elements related to a particular disease process from cross-sectional studies requires prohibitively large cohorts. Alternatively, implementation of systems biology principles to interventional clinical trials represents a unique opportunity to gain predictive understanding of complex diseases in comparatively small cohorts of patients. This paper reviews systems biology principles applicable to translational research, focusing on lessons from systems approaches to inflammation applied to multiple sclerosis. We suggest that employing systems biology methods in the design and execution of biomarker-supported, proof-of-principle clinical trials provides a singular opportunity to merge therapeutic development with a basic understanding of disease processes. The ultimate goal is to develop predictive computational models of the disease, which will revolutionize diagnostic process and provide mechanistic understanding necessary for personalized therapeutic approaches. Added, biologically meaningful information can be derived from diagnostic tests, if they are interpreted in functional relationships, rather than as independent measurements. Such systems biology based diagnostics will transform disease taxonomies from phenotypical to molecular and will allow physicians to select optimal therapeutic regimens for individual patients.

## Introduction

Complex polygenic diseases, whether inflammatory or degenerative, represent significant societal problem (1). Multiple sclerosis (MS) is polygenic inflammatory disorder of the central nervous system (CNS). Its early, relapsing–remitting stage (RR-MS) is characterized by aberrant immune responses causing demyelination and axonal damage and is successfully treated with immunomodulatory therapies. Later, progressive stages of the disease do not respond to immunomodulatory treatments. Because initiation of progressive phase of the disease is related to the patient’s age more strongly than to parameters of previous disease activity, it has been considered to represent an accelerated neurodegenerative process. Pathology studies suggest that despite causal, genetic, and phenotypical diversity, surprisingly similar pathogenic processes (e.g., inflammation, mitochondrial dysfunction, oxidative stress, endoplasmic reticulum (ER) stress, excitotoxicity, DNA damage, autophagy, and tissue remodeling) are observed in the target tissue of patients with varied polygenic diseases (1, 2), including different stages of MS (3). However, it is not clear whether these processes are irrelevant bystander effects of one dominant (i.e., disease-specific) mechanism, or whether they evolve, interact (4), and contribute causally to the development of disability. This ambiguity has stymied the development of effective, potentially curative disease modifying treatments (DMTs).

Polygenic diseases also represent extraordinary scientific problem, because their genetic heterogeneity, multifaceted pathophysiologies, and complex environmental influences are difficult to reproduce in experimental conditions. Systems biology is a compendium of scientific methods complementary to the use of pre-clinical experimental models, since pre-clinical models invariably represent simplified versions of the actual disease process (5–7). The addition of systems biology approaches to traditional investigatory procedures is poised to accelerate understanding of the causative disease mechanisms (8). Herein, however, we argue that such advances require not only the use of systems biology approaches on the part of basic scientists, but also the utilization of such methods in clinical trial methodology (9–12) and eventually, as our understanding increases, also in clinical practice.

## Reconfiguring Reductionist Research with Systems Biology Approaches

**Figure 1:**
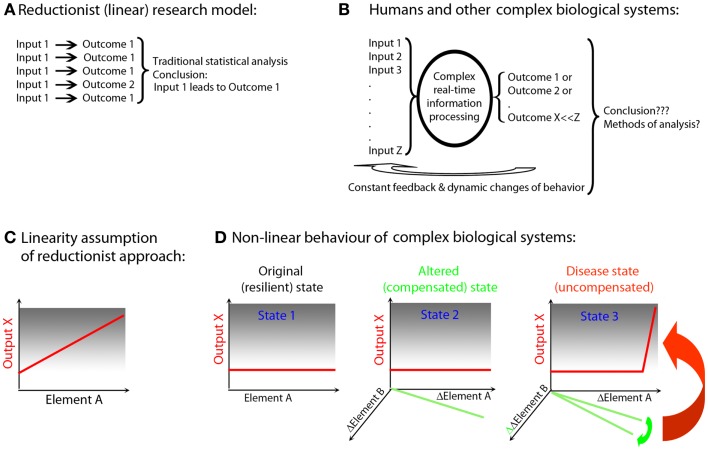
**Simplified animal models versus complex biological systems exemplified by human polygenic diseases**. **(A)** Reductionist (linear) research model (e.g., experimental autoimmune encephalomyelitis): current animal studies are almost exclusively performed in a single animal species of a single genetic strain (usually the one that is susceptible to induction of the disease). Furthermore, the animals are housed in the same (often pathogen-free) environment; they are exposed to identical food and identical environmental stimuli, which leads to synchronization of circadian rhythms, similar levels of activity, etc. Disease is induced by identical regimens applied in a highly synchronized manner to animals of the same age and often only of single sex. Therefore, animal experiments utilize highly simplified input (input 1). Despite standardized input, the outcome is usually somewhat heterogeneous (outcome 1 and 2), but an application of traditional statistical methods leads to clear conclusions. These conclusions are often readily generalized across species and across diverse environmental inputs and disease triggers. **(B)** Humans and other non-artificial complex biological systems: measurements in the complex biological system exemplified by a human being affected by a disease are the results of multiple different inputs (i.e., an outbred genetic background, many environmental influences such as type, dose, and virulence of an infectious agent, diverse food, premorbidities, and drug regimens influencing both the metabolome and the microbiome, varied endocrine regulations resulting from circadian and reproductive rhythms and aging). The organism processes these varied inputs, utilizing complex decision-making mechanisms and the outcomes are also diverse (e.g., maintenance of heath or development of the disease of varied severity). Furthermore, the outcomes are processed by the organism as additional inputs through constant ubiquitous feedback loops, leading to dynamic changes of behavior. Current statistical methods are largely inadequate for analysis of such complex datasets. As a consequence, frequently no reproducible conclusions are reached. **(C)** Linearity assumption of reductionist approach: reductionist research methods are based on assumption that if an element (e.g., gene, its transcript or protein; Element A) is linked to a disease process (output X), then an observed or induced change in the element has to be reflected in the change in the output. **(D)** Non-linear behavior of biological systems: the existence of functional networks consisting of interacting elements with partially overlapping functions allows biological systems to retain a given output in a normal (non-disease) state if only one or few elements have been altered (this is called robustness of the system). For example, an alteration in Element A was compensated by a reciprocal change in Element B (State 2). However, despite maintenance of the same output, this new state is different from the original state (healthy; State 1), even though phenotypically there is no evidence of a disease. Such an altered state of the system is more fragile, insofar as any subsequent change in Element B may now cause a dramatic change in the output X (i.e., disease state; State 3).

The genetic and environmental diversity of the human population leads to a vast, heterogeneous “input” into disease states. This tremendous heterogeneity stands in direct contrast to the homogeneity of simplified pre-clinical models, such as single genetic mutations expressed on an identical genetic background of inbred animals housed in an artificially controlled environment (13) (Figure 1), and often studied at very few time points. Elimination of “biological noise” by unnaturally limiting genetic, environmental, age-related, and reproductive diversity allows isolation of the mechanism(s) related to the studied process and underlies the traditional success of widely utilized reductionist research methods. However, reports that pathogenic mechanisms may differ based on the genetic background of the animal (14), or that variations in bacterial flora influence the development of pathogenic immune responses (15, 16), question general applicability of findings obtained in the simplified pre-clinical paradigms. If genetic pre-disposition is insufficient for the development of polygenic diseases such as MS (17), then the elimination of environmental influences (18, 19) that may interact causally with inherited susceptibility alleles is a type of reductionism that is likely to be detrimental to our understanding of the true disease process. Simplification may indeed be necessary to discern disease mechanisms, but it has to be simplification driven by rational means, not by convenience. Data-driven simplification, i.e., extraction of “essential elements” from multimodal datasets, and modeling how these elements interact *in vivo*, is the basis of systems biology (5–7, 20) and could actually inform development of better pre-clinical models.

A further problem of reductionist research lies in the extrapolation of inferred linear, causal relationships between an identified biological Element “A” (e.g., gene, mRNA, protein) and the manifestation of a system-level, phenotypic output X (e.g., disease activity) (Figure 1C). Through evolution, organisms have developed progressive complexity and redundancy via duplication and adaptation of existing genes (21, 22) that can maintain functions of the organism within physiological ranges, ranges which are resilient to alterations of a single or even multiple molecules. The elements of the system that interact and are able to compensate for each other’s function form the basis of an *in vivo* functional network (10, 23) or subsystem (24). Thus, a change in Element A may be compensated for by a reciprocal alteration in Element B to keep output X unchanged (Figure 1D, State 2). Without monitoring changes in all interacting elements (which requires either measurements of all components of the system or pre-existing knowledge of which elements constitute the relevant subsystem), one may come to a false conclusion about causal relationships. In the example utilized above, Element A is still causally related to output X, because the new “homeostasis” resulting from adaptation of the Element B makes this subsystem more fragile, i.e., susceptible to failure with the next alteration. Accordingly, even a small, successive change in Element B, which would have been easily compensated for if the functional network was in its original, resilient state, may now lead to a robust change in the output X (Figure 1D, State 3). This new state is characterized by the failure of control over this particular functional network, expression of which is the disease.

This example also explains why there are often only mild differences in causally related elements observed between individuals with and without a disease. Absent a disease process, the controls will include subjects in States 1 and 2 (i.e., with normal and adapted Elements A or B, who are nonetheless able to compensate functionally). In contrast, patients with the disease will exhibit alterations in Elements A and B that have exceeded the compensatory potential of the network. Not surprisingly, we could expect greatly overlapping values in Elements A or B when each of them is measured in isolation. If studied cohorts are large enough, we may be able to discern statistically significant differences between controls and patients utilizing standard statistical measures. However, we will unavoidably underestimate the strength of the causal relationship for each element because of the variance introduced by the variable levels of network compensation. From genetic to functional studies, we have all experienced this predictable plight that results from the application of reductionist methodology to non-linear biological systems (25). However, if data are integrated non-linearly (in real life or in a computational simulation), what appear to be trivial differences among individuals may lead to vastly different outcomes, depending on the initial conditions of the system.

We noted before that we can avoid making false conclusions about causal relationships if we measure all parameters in the system (which is practically impossible), or if we simultaneously quantify elements belonging to the same module. We will suggest later how the use of clinical trials facilitates the identification of elements pertaining to the same functional network *in vivo*, emphasizing the importance of capturing data obtained upon perturbations of the system.

Although real networks are undoubtedly more intricate than the example utilized in Figure 1, the described theoretical concept can be encountered in clinical practice. For example, daclizumab, a monoclonal antibody (Ab) that blocks formation of high affinity IL-2 receptor, effectively reduces MS disease activity (26, 27) (output X), while inhibiting FoxP3^+^ T-regulatory cells (T-regs; Element A)(28, 29). Paradoxically, FoxP3 T-regs are the best known cell type that prevents development of systemic autoimmunity (30). If interpreted in a reductionist way, the above observation would lead to the conclusion that in contrast to other autoimmune conditions, T-regs do not play an immunoregulatory role in MS and may, in fact, be detrimental. Nonetheless, this conclusion is likely incorrect, because daclizumab also activates another regulatory cell population, CD56^bright^ NK cells (31) (Element B), cells that belong to the same *in vivo* functional network as T-regs (and effector T cells) based on their competition for IL-2 (29). CD56^bright^ NK cells have overlapping immunoregulatory functions with T-regs, although NK cells carry out different regulatory programs (i.e., limiting T cell expansion by granzyme-K-mediated cytotoxicity) (32) and may be especially important for regulation of intrathecal inflammation (33–35). The new steady state induced by daclizumab is clearly beneficial for MS. However, this state is nevertheless an alteration of the normal “healthy” state, and thus may be less resilient (36). In fact, increased skin inflammation was observed during daclizumab treatment (26, 28), which may be related to inhibition of T-regs. Moreover, a single patient who failed to expand CD56^bright^ NK cells during daclizumab treatment developed CNS vasculitis (37), which is perhaps an example of a non-linear consequence of further alteration of the targeted functional network (Figure 1D, State 3).

## Evolution of Systems Thinking to Systems Biology

**Figure 2:**
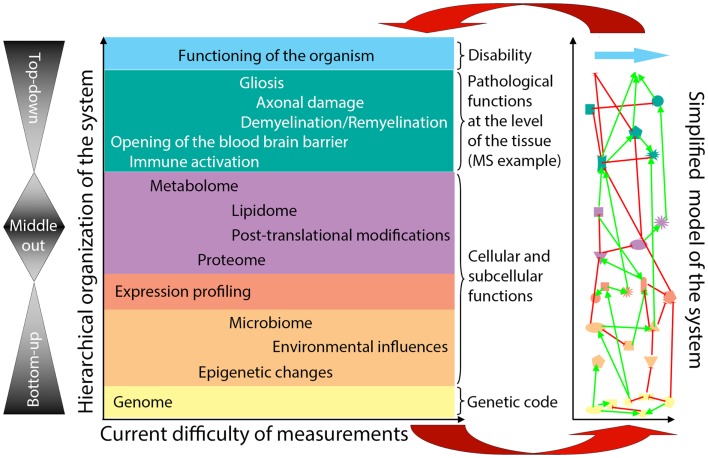
**Organizational hierarchy of the human body affected by the disease (example of MS) and its simplified model**. Schematic illustration of the organizational layers (depicted by different colors) of human body affected by MS. *X* axis depicts relative ease (left) versus difficulty (right) of obtaining high-quality quantifiable data within each organizational plane. The panel to the left of the organizational chart represents different methodologies utilized by systems biology: “bottom-up approach” uses genomic data to predict disease occurrence or severity. “top-down” divides patients into diagnostic categories and analyzes disease-specific differences in the transcriptome by expression profiling of accessible tissues, such as blood; “middle-out” approach (probably the least utilized, while most useful) collects and analyzes data within one organizational plane and then expands data gathering vertically in both directions: e.g., one can start by analyzing data on the cellular level and expand these analyses downward by expression profiling of studied cells and upward by pathological studies of the affected tissues. The panel to the right of the organizational chart represents an idealized model of the studied system that emerges from a systems biology approach. In this simplified model, each organizational level is represented by limited number of essential elements, which are derived from multiple different studies (e.g., simplified *in vitro* models, including those derived from iPS cells, organotypic cultures, animal models and finally, human observational, and interventional studies) that provide information that is relevant to the particular disease entity, in our case MS. The interactions (protocols) among the essential elements are schematically depicted as different types of connecting lines (with the understanding that protocols represent complex behaviors that go beyond currently utilized positive or negative interactions). For example, on the genomic (yellow) level, essential elements are known MS susceptibility alleles, as well as yet unknown genetic variants that modulate MS disease activity or phenotype. On cellular/tissue level (green), essential elements are all cells of the immune system that mediate damage (e.g., cytotoxic T cells) or promote repair (e.g., regulatory immune cells, alternatively activated macrophages), as well as cells of the CNS that are the target of inflammation (e.g., oligodendrocytes and myelin) or the source of healing (e.g., oligodendroglial precursors in remyelination and neural stem cells in adult neurogenesis). Thus, each organizational level can be said to represent a “system.” There may be further subsystems within organizational levels, such as, e.g., myelination within cellular/tissue system. Yet, there are also vertical interactions among individual organizational levels, making a disease like MS a “complex system” as defined by Mesarovic et al. (24). For example, the genetic background of the individual together with acquired epigenetic changes and microbial influences present at the time of disease induction determine the severity of intrathecal inflammation, the ability of CNS tissue to survive inflammatory insults and the forms and levels of repair. The essential elements at all hierarchical levels form an “*in vivo* functional network” and the interactions among these elements capture the essence of the information processing and output of this functional network [i.e., different protocols or scripts regulating behavior of each subsystem and the interactions between them (36)]. The model itself is constructed by integration of data analyses from different studies of the disease process, as well as varied physiological processes (e.g., MS in its entire evolution from early relapsing-remitting disease to late secondary-progressive MS, but also studies of developmental myelination or basic immune regulation). Until a complete understanding of a disease process is achieved, the corresponding model always represents a “work in-progress.” Models develop as each new study adds or validates information about essential elements or about protocols within individual subsystems or about interactions between subsystems that affect behavior of the system. The value of this simplified model resides in its ability to predict the behavior of the biological system based on the input data. The validity of the model has to be determined by repeated confirmation of model-based predictions in the studied biological system *in vivo* (upper red arrow). Paradoxically, discordant observations are the most valuable for guiding further efforts to adjust the computational or conceptual model (lower red arrow), leading to an increasingly better understanding of the system.

General systems theory (21, 38) has been a long-established academic discipline based on the axiom that “a system is more than the sum of its parts” (21). Incorporation of systems thinking into biomedical research is being driven by the challenge of integrating large datasets of “omics” data (39–42) in a search for organizing principles that underlie biological functions (24). Considering the hierarchical organization of a complex biological system (e.g., the human body affected by MS), there are considerable differences in our ability to quantify elements at each organizational level (Figure 2, *X*-axis). Because of the ease of measurements obtained at the genome level and the determination of gene expression in accessible tissues, initial “omics” studies strived to directly correlate the bottom layers of the hierarchical organization with its very top. However, due to extensive subcellular and cellular levels of regulation, only part of the information captured in lower hierarchical layers directly influences functioning of the organism. One consequence of this uncoupling is the prohibitively large sample cohorts that are required in order to identify disease-relevant elements from lower hierarchical levels in cross-sectional cohorts (25), as demonstrated convincingly in genomic studies.

Intriguingly, systems theory determined that individual elements are much less important determinants of the behavior of a given system, as compared to interactions among these elements (24, 38). Thus, defining interactions among the elements of a complex system [otherwise called “protocols” (36)] is expected to provide a deeper understanding of the system than simply cataloging system parts (13, 24). Indeed, understanding the operational protocol(s) in one module may provide predictive understanding of another module, because the robust organizing principles are evolutionarily conserved and re-used in different functional networks (24). For example, the knowledge about the ability of IL-15Rα expressed on one cell to *trans*-present IL-15 to another cell (43) informed the discovery that IL-2Rα (CD25), which is closely related to IL-15Rα, can also present IL-2 to different immune cells *in-trans* (44).

While first hints about interacting elements can be derived from their association (e.g., correlations) observed *in vivo* (10), a full understanding of the nature of relationships among individual elements requires functional assays that are generally laborious, expensive, and difficult to standardize. Such assays can be applied only to a limited number of samples. We will argue that implementing such mechanistic studies into Phase I/II clinical trials, in conjunction with systems biology approaches, poses considerable advantages in comparison to the attempts to gain mechanistic insight from cross-sectional cohorts.

## Systems Biology Workflow for Translational Research

Ideker et al. in their seminal paper (5) described a general systems biology workflow of four steps.

### Define the components of the system

Some level of understanding of the components and the organizational structure of the system is necessary for formulation of initial hypotheses about how the system operates. If the critical elements of the studied system are unknown, we need to acquire high-quality data spanning multiple hierarchical levels. The inability to collect complete datasets should not dissuade us from implementing a systems biology approach. Rather, we should recognize that the development of an accurate model – be it conceptual or computational – of a given disease is an iterative process that will require multiple rounds of adjustments to hone in on key biological mechanisms (45). Standardized collection and storage of biological samples may bridge the present inability to measure data comprehensively by exploiting future methodological advances. What is essential, however, is that in addition to sample collection for cataloging “elements,” we start implementing functional and mechanistic studies that reveal “protocols” (36).

### Systematically perturb the system and monitor its components in order to identify *in vivo* functional networks and develop first models of the system

Interventional experimentation, employing biological, genetic, or chemical manipulations, is vital to basic science. In contrast, translational scientists strive to gain insight primarily through observational studies. Such a strategy is extraordinarily ineffective, for the reasons outlined previously.

**Figure 3:**
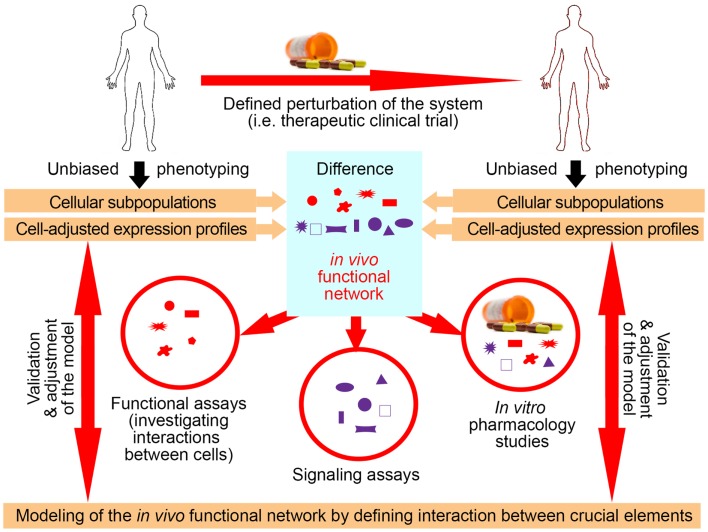
**Utilization of clinical trials with associated biomarker studies to increase understanding of the biological system**. Clinical trials represent a unique opportunity for employing systems biology research methods. Data are obtained on multiple organizational levels before and after treatment from the same set of patients. Subtraction analysis (i.e., comparing within each subject data collected before and at specified time-intervals after initiation of treatment and considering those markers that change synchronously in subjects sharing the same treatment allocation) then identifies those changes that are *direct*, or, more often, *indirect* consequences of applied therapy. While applied therapy affects some elements directly (for example rituximab directly depletes B cells), this primary effect induces multiple secondary effects, as the system “adjusts” to the induced change. For example, inhibition of certain T cell functions under rituximab therapy would “alert” the investigators to the fact that T and B cells normally interact *in vivo* in mediating antigen-specific immunity, because the lack of B cells has to directly or indirectly explain the observed functional deficit in T cells, as T cells do not express CD20, the target of rituximab. Of course, it was known before initiation of rituximab trials that T and B cells interact together, but by analyzing samples from clinical trials of daclizumab we gained new insights into elements of the immune system that had previously been obscure such as innate lymphoid cells and their unexpected role in the MS disease process. Elements that are changing synchronously by applied therapeutic perturbation are part of the same “*in vivo* functional network” that has been disturbed by treatment. Identification of gene transcripts, proteins, and cell types that interact with each other *in vivo* then greatly facilitates investigation of interactions between these elements in simplified models, such as *in vitro* functional assays, *ex vivo* signaling assays, or *in vitro* pharmacology studies. Mechanistic insight (i.e., identified protocols) gained from these simplified models has to be verified *in vivo*, either by studying a new set of patients or by applying new therapies.

Utilization of exogenous perturbations provides an opportunity to collect multi-modality data from the same human being before and in specified time period(s) after application of the stimulus that perturbs the system in a highly standardized manner (Figure 3). Because each subject serves as his/her own control, such a paradigm limits influences of genetic and environmental diversity and allows scientists to identify those components of the system that were specifically affected by the perturbation. Such an approach represents a unique opportunity to identify key components of the system that interact *in vivo*, i.e., that are part of the same functional module.

Identification of the elements that belong to the same module then radically facilitates mechanistic studies aimed at defining protocols that regulate the behavior of the module (Figure 3), because such studies can now focus only on identified components. This concept is again exemplified in clinical trials of daclizumab in MS, which were heavily supported by mechanistic studies (26, 29, 31, 34, 44, 46). For example, observations that daclizumab therapy causes coordinated changes in the numbers of activated T cells and CD56^bright^ NK cells (31) prompted an intense search for the protocols that guide interactions between these two cell types (32). This led to the discovery of the granzyme-K-mediated cytotoxicity of CD56^bright^ NK cells toward autologous activated T cells, despite the fact that CD56^bright^ NK cells were thought to have low cytotoxic potential (47) and the prevailing immunological dogma stipulated that NK cells cannot kill autologous, MHC-I expressing cells. Despite controversy raised by the publication of human studies, physiological NK-mediated killing of activated autologous T cells has now been decisively confirmed in animal systems (48). Similarly, it was the observation that daclizumab therapy causes reciprocal expansion of CD56^bright^ NK cells and contraction of pro-inflammatory lymphoid tissue inducer (LTi) cells that incited search for the functional relationship between these cell types. Benefiting greatly from published animal studies (49, 50), we were able to rapidly formulate and mechanistically confirm a unifying hypothesis about developmental association between CD56^bright^ NK cells and LTi cells in humans, where intermediate affinity IL-2/IL-15 signal plays a decisive role in promoting differentiation of common innate lymphoid cell (ILC) precursors away from LTi and toward NK cell lineage (46). These two examples demonstrate how, in conformity with systems biology workflow, incorporation of biomarker, and mechanistic studies to small (Phase I/II) clinical trials generated unexpected insight about human functional network that contains elements not previously linked to MS disease process (i.e., CD56^bright^ NK cells and LTi cells), but with possibly high pathophysiological potential (33, 47, 51).

Thus, although the only widely available perturbations that can be utilized in humans are exogenously applied therapies within the context of clinical trials, we should not view this as a limitation. Rather, clinical trials, especially in their earliest stages (Phase I/II) embody a unique opportunity in this regard: first, the clinical infrastructure is already assembled and the acquisition of clinical, laboratory, and imaging data is much denser than in clinical practice. Therefore, highly standardized multi-modality data are already collected as part of clinical trials, and thus in general no additional investments are required. Second, these trials are often performed in leading academic centers that have a broad research infrastructure, which can be employed for collection and processing of biological samples and, most importantly, for implementation of targeted functional assays. While this step does require additional resources, the investment is remarkably smaller than situations in which sample collection and functional assays were not coupled to a clinical trial, and the entire infrastructure for systems biology studies would have had to be created *de novo*. Additionally, because only a minority of treatments proceed to Phase III testing, focusing on Phase I/II trials offers an advantage for studying a broad array of perturbations, including those that will fail (11) and therefore go against a current conceptual model of the disease. Indeed, such “failures” hold the potential to dramatically increase knowledge through the necessary revision of the disease model (45, 52). For example, if biomarker measurements in treated patients demonstrated that the drug had the predicted effect on a particular molecular mechanism in the target tissue, but no efficacy on tissue destruction was observed, we would then be able to conclude that the targeted molecular process does not contribute directly to the higher-order phenomenon at the tissue level. Such a “negative” trial would still significantly advance the field.

The inclusion of systems biology (e.g., modeling) as a core part of the clinical trial process also poses great advantages for drug development. Accompanying biomarker studies may not only determine whether or not the candidate drug has the expected mechanistic effects *in vivo*, but could also guide rational dose selection for subsequent Phase III trials, generate candidate biomarkers that either predict or reflect therapeutic efficacy (31), and optimize the target patient population [by identifying subjects with a therapeutic target and/or screening out subjects with a pre-disposition to side effects (53)]. Such candidate biomarkers may be then validated seamlessly in Phase III trials, as is currently being done for CD56^bright^ NK cells as a biomarker of therapeutic efficacy of daclizumab therapy in MS (27, 31).

Although the inclusion of systems biology methods in clinical trials requires additional resources, the combined benefits of this approach for the sponsor (i.e., lower cost of Phase III trials due to optimal selection of therapeutic dose and patient population, potentially joint biomarker/drug regulatory approval leading to post-marketing advantage), for the patients/society (i.e., enhanced risk/benefit stratification, avoidance of dosing subjects that lack the therapeutic target), and for scientific community (i.e., obtaining critical mechanistic insight into the disease process) should provide an impetus for all interested parties to advocate, search for, and find creative solutions to the funding problem.

### Reconcile the experimentally observed responses with those predicted by the computational models

A key concept is that the more complex the system is, the more difficult it is to determine whether any model truly predicts the behavior of the system (38, 45). From the standpoint of model refinement, the failure of model predictions – rather than its successes – paradoxically carries more information for an eventual understanding of the system (7, 21). Concordance of the behavior between the model and the system tells us only that the model faithfully predicts functioning of the system *within tested parameters*. Discordance, on the other hand, indicates that our understanding of the system is either incorrect or insufficient and requires modification. For example, efficacy of immunomodulatory therapies in RR-MS, including long-term efficacy of high dose immunosuppression followed by autologous bone marrow transplantation (aBMT) (54), if applied to young patients with short disease duration, provide strong evidence for the immune-driven pathogenesis of RR-MS. On the other hand, failure of the same therapeutic modalities to stop accumulation of disability in progressive MS, while retaining its high efficacy on MRI contrast-enhancing lesions (CEL) (54, 55), provides a basis for the currently broadly accepted view that neuro-degeneration, rather than inflammation drives disability in progressive MS. While this is certainly a possibility, it represents only one of several possible explanations. We assume that therapeutic modalities such as alemtuzumab or high dose immunosuppression followed by BMT successfully inhibit all intrathecal inflammation in progressive MS based on their efficacy on CEL. However, rare instances when the level of remaining CNS inflammation was quantified directly (i.e., by pathology studies after patient’s death) suggests otherwise (56) and point to the fact that our assumptions are often simplistic representation of complex reality. While CEL may indeed be caused by dense perivascular infiltrates in some RR-MS patients, they cannot be viewed as a biomarker of all types of intrathecal inflammatory activity. Therefore, rather than committing to the first, perhaps most obvious interpretation, systems biology approach searches for all possible explanations of a given observation and utilizes a new perturbation experiment to distinguish between alternatives.

### Design and perform new perturbation experiments to distinguish between multiple and competing model hypotheses and to provide additional data for improvement of the model

Clearly, a single experiment embodied by a single interventional trial will not provide a complete understanding of a given disease. Rather, different clinical trials will generate partially overlapping, but mostly complementary information that, when integrated on the bioinformatics level (11, 45), can ultimately reveal the true nature of the disease process in a manner analogous to how assembled pieces of the jigsaw puzzle reveal the underlying image. Although such bioinformatics-based integration of information derived from different clinical trials has not been performed yet in MS, the closest existing example we could think of is the Bayesian-based design and analysis of the I-SPY-2 TRIAL (Investigation of Serial Studies to Predict Your Therapeutic Response with Imaging and Molecular Analysis 2; www.ispy2.org). This innovative clinical trial randomizes patients with newly diagnosed breast cancer to different treatment arms based on the analysis of patient-specific tumor biomarker profiles in relationship to accumulating knowledge from the ongoing analyses of outcomes of previously enrolled patients. Thus, each patient’s molecular tumor signature is preferentially paired with the type of chemotherapeutic agent that previously showed the best efficacy for that particular type of tumor. The efficacy data are processed and incorporated in real time to generate an optimized predictive scheme for the next set of enrolled patients. At the same time, the outcomes from different studied treatments are compared to each other so that ineffectual or toxic drugs can be abandoned and substituted with novel agents. I-SPY-2 TRIAL can be viewed as a compendium of multiple trials, which all benefit from the shared infrastructure and know-how (biomarker analyses, mathematical modeling), providing societal value way beyond determination of the efficacy of a single agent. The trial is sponsored by the Biomarker Consortium, a unique partnership between the Foundation for NIH (FNIH) and a large number of pharmaceutical companies, academic medical centers, and patient advocacy groups. As such, it represents the prime example of the creative funding solution(s) we advocated for in the previous section.

Given the scope and complexity of the systems under study, integration of knowledge from different sources will almost certainly require both biological (e.g., *in vitro* experiments with immune cells or iPS cells differentiated in CNS cell subtypes, as well as enhanced animal models) and mathematical modeling [e.g., *in silico* experimental workflow (57)]. Investigators can then draw on these data and the correlations derived from bioinformatics analyses both to better understand causal mechanisms and to guide the selection and design of future interventions.

## From Systems Biology to Personalized Medicine and Beyond

Although in this paper, we advocate implementation of systems biology methods to clinical trial methodology as a tool to gain predictive understanding of disease processes, we need to acknowledge that methods of systems biology will have much broader application to all of clinical medicine. Systems biology-derived informatics that goes beyond the current paradigm of statistically based bioinformatics (53, 58), are an absolute prerequisite for personalized or precision medicine (13). For example, although we currently utilize in clinical practice a large number of validated laboratory measurements, we judge the abnormality of these measurements independently of each other. However, they do not represent independent values, as many of them actually belong to shared functional modules. If instead, we applied an understanding of the protocols that underlie relationships between these dynamic biomarkers in a living system to interpretation of laboratory results (i.e., by using all simultaneously obtained laboratory measurements as “input” into a computational model of human homeostatic regulation) we would obtain information that greatly exceeds current designation of “normal” or “abnormal” results. The predictive model should be able to pin-point laboratory error (if obtained values in one biomarker are incompatible with obtained values in remaining biomarkers that are part of the same functional network) or specify the type of homeostatic failure that is capable of producing the obtained results. We have little doubt that such methodology will be the basis of future diagnostic processes.

Furthermore, once we can model diseases computationally as an integrated, ongoing, and evolving process, we will also gain the ability to more effectively treat individual patients. Due to advances in molecular diagnostics, it is now well-appreciated that defects in different genes can lead to phenotypically similar disease expression (59). Similarly, many neurodegenerative diseases have both polygenic (common) and monogenic (rare) disease variants. Indeed, failure of the regulation of one functional network can have multiple different causes and it is likely that optimal therapies may likewise differ depending on causal element(s). Validated molecular, cellular and functional biomarkers will be able to pin-point specific types of regulatory failure that underlies disease expression in a particular subject from whom these measurements were obtained. For example, we envision that in the not so distant future, development of new, more sensitive, and cell-specific biomarkers of intrathecal inflammation will be able to select those patients with progressive MS who will benefit from novel immunomodulatory therapies. Such biomarkers will also facilitate development of these novel treatments and serve as guidance for the treating clinician who is monitoring their efficacy in clinic. Thus, clinicians will no longer think about “old” categories of diseases, but instead about molecular signatures that define new disease taxonomy (58), about dysregulated pathways, stochastic processes, and failed functional networks (8). We will gain individualized prognostic insight, and, above all, we will be able to rationally select and optimize treatment combinations for this particular patient.

## Conclusion

Systems biology is not an approach in which the mindless application of powerful technologies can compensate for the lack of creative thinking. Rather, the integration of systems thinking with dynamic computational modeling can lead to the development of a “virtual sandbox” in which researchers can utilize their creativity and intuition to try out and explore multiple different hypotheses and lines of investigation. From creative design of clinical protocols, accompanying functional assays and computational algorithms for data analyses, to imaginative data integration and reduction (60), thoughtful adaptation of knowledge from previously identified protocols to new regulatory modules and biological systems, it is clear that success of systems biology requires pioneering visionaries as much as highly collaborative teams. While technical developments provide opportunities, conceptual advances are the true drivers of the progress.

## Conflict of Interest Statement

Dr. Bibiana Bielekova is co-inventor on NIH patents related to daclizumab therapy and as such has received patent royalty payments from NIH. Remaining authors have nothing to disclose.
